# Transcriptomic Analysis of Starvation on the Silkworm Brain

**DOI:** 10.3390/insects14070658

**Published:** 2023-07-24

**Authors:** Yi Li, Xin Wang, Haonan Dong, Qingyou Xia, Ping Zhao

**Affiliations:** Integrative Science Center of Germplasm Creation in Western China (CHONGQING) Science City, Biological Science Research Center, Southwest University, Chongqing 400715, China; yili89716@gmail.com (Y.L.); swuwangxin@swu.edu.cn (X.W.); haonandong.1995@gmail.com (H.D.); xiaqy@swu.edu.cn (Q.X.)

**Keywords:** starvation, silkworm, brain, transcriptome

## Abstract

**Simple Summary:**

To shed light on the influence of starvation on animal brains, a comprehensive transcriptome analysis was carried out on silkworms. The subsequent enrichment analysis unveiled noteworthy changes in pathways associated with tissue structure, hormone metabolism, longevity, nucleic acid metabolism, immune response, and disease within the silkworm brain after the period of starvation.

**Abstract:**

Starvation imposes significant stress on animal survival and development, resulting in organ damage within the organism. The brain, being one of the most vital organs in animals, plays a crucial role in coordinating the physiological functions of other organs. However, performing brain experiments on the human body is challenging. In this work, we selected the silkworm, a model Lepidoptera organism, due to its favorable characteristics. A comprehensive transcriptome analysis was conducted on the brain of silkworm subjected to starvation treatment. The analysis of differentially expressed genes revealed significant alterations in 330 genes following the period of starvation. Through an enrichment analysis, we successfully identified pathways associated with metabolism, hormones, immunity, and diseases. Our findings highlight the transcriptional response of the brain to starvation, providing valuable insights for comprehending the impact of starvation stress in other animals.

## 1. Introduction

According to the World Health Organization (WHO), starvation stands as the most severe and pressing threat to global public health. Throughout their lifetimes, most species undergo sporadic episodes of starvation [1], with the duration of the food shortage dictating the physiological mechanisms at play. Prolonged starvation inflicts damage upon the body, culminating in eventual demise. Although short-term starvation does not impose significant harm upon animals, it nonetheless elicits a multitude of reactions within the organism. 

The gut, which plays a vital role in the digestive system, is often referred to as the “second brain” in animals [2]. Recent research has provided compelling evidence of the profound impact of starvation on the animal gut [3]. Among children afflicted with severe acute malnutrition, there is a significant decline in epithelial surface area and enterocyte cell mass [4]. Experimental animal models subjected to starvation exhibit noteworthy decreases in intestinal weight, villus surface area, and enterocyte mass, coupled with an increase in intestinal permeability [5,6]. Furthermore, in patients diagnosed with anorexia nervosa, starvation exerts an influence on the microbiome and the intricate interaction between the gut and the brain [7].

The brain functions as the central control center for animals and is vulnerable to the consequences of starvation. In human studies, researchers investigated the cerebral metabolism of glucose and ketone bodies in nine healthy volunteers both before and after a 3.5-day period of starvation. The findings revealed a reduction in glucose metabolism within the brain [8]. Additionally, single-cell sequencing performed on the *Drosophila* brain unveiled changes in the cellular composition and gene expression profiles of larval brains induced by starvation [9]. In *Drosophila*, starvation also influences neuronal histone modifications [10].

Conducting starvation experiments on the human body, particularly when analyzing the gene expression profile of the brain, presents significant challenges [11]. Insects, however, offer a convenient research subject as they can be easily reared without ethical constraints. Previous studies have demonstrated the utility of insect brains as a model for investigating aging processes [12]. Among insects, the silkworm stands out as an exemplary representative of the Lepidoptera order and has been extensively employed as a valuable model organism in various research domains, including human disease research, screening for antimicrobial agents, environmental safety monitoring, and antitumor studies [13,14,15]. Therefore, in this study, we utilized silkworms to analyze the gene expression profile of the brain following starvation treatment. The findings from this research hold the potential to provide valuable insights into the effects of starvation on animal brains.

## 2. Materials and Methods

### 2.1. Insect

The silkworm eggs, strain Liangguang 2, were purchased from Guangtong Silkworm Eggs Company (Qingzhou, Shandong, China). Eggs were incubated at 28 °C with 75% humidity until larvae hatch. Subsequently, the silkworm larvae were nourished with fresh mulberry leaves until the 1st day of 5th instar (I5D1).

### 2.2. Starvation Treatment and RNA Preparation

The larvae at I5D1 were divided into two groups, each consisting of 60 individuals. The control group was fed with mulberry leaves as per normal, while the treatment group was subjected to the same conditions but without food for a duration of 24 h. Following the treatment period, each individual was dissected under a Zeiss microscope, and their brains were carefully collected. Total RNA extraction was performed using TRIzol Reagent (ThermoFisher, Waltham, MA, USA) following the manufacturer’s instructions. The RNA concentration, purity, and integrity number were assessed using the Agilent 2100 Bioanalyzer.

### 2.3. Library Construction and Illumina Hiseq Xten Sequencing

The RNA-seq library was prepared using the TruSeqTM RNA Sample Preparation Kit (Illumina, San Diego, CA, USA), with 1 μg of total RNA as input. To isolate messenger RNA (mRNA), polyA selection was performed using oligo(dT) beads, followed by fragmentation using a fragmentation buffer. The Super-Script Double-Stranded cDNA Synthesis Kit (ThermoFisher, Waltham, MA, USA) with random hexamer primers (Illumina, San Diego, CA, USA) was utilized for the synthesis of double-stranded cDNA. The synthesized cDNA underwent end-repair, phosphorylation, and ‘A’ base addition, following Illumina’s library construction protocol. Size selection of the libraries was conducted using 2% Low Range Ultra Agarose to obtain cDNA target fragments of 300 bp, and PCR amplification with Phusion DNA polymerase (NEB, Ipswich, MA, USA) was carried out for 15 cycles. The quantification of libraries was performed using the TBS380 (TurnerBio-Systems, Sunnyvale, CA, USA), and the paired-end RNA-seq sequencing library was sequenced using the Illumina HiSeq xten sequencer with a read length of 2 × 150 bp.

### 2.4. Read Mapping

The raw paired-end reads were subjected to trimming and quality control using SeqPrep and Sickle tools, utilizing default parameters. Following this, the resulting clean reads were aligned individually to a reference silkworm genome assembled by our laboratory (BmDZ.v3.6, https://silkdb.bioinfotoolkits.net/main, accessed on 1 August 2022) using HISAT2 (2.1.0) software [16] in the orientation mode. The mapped reads from each sample were then assembled using StringTie in a reference-based approach [17].

### 2.5. Differential Expression Analysis

To determine the differential expression of genes (DEGs) between two distinct samples, the expression level of each transcript was quantified using the transcripts per million reads (TPM) method [18]. RSEM (1.3.3) [19] was used to quantify gene abundances. In essence, differential expression analysis was conducted utilizing the DESeq2 (1.24.0) algorithm [20]. Genes with |log2FC| > 1 and Q value <= 0.05 were deemed as significantly different expressed genes.

### 2.6. Functional Annotation and Enrichment of DEGs

To analyze the biological function of DEGs, COG (Clusters of Orthologous Groups), GO (Gene Ontology), and KEGG (Kyoto Encyclopedia of Genes and Genomes) annotation were performed by eggNOG-mapper (http://eggnog-mapper.embl.de/, accessed on 1 October 2022) [21] with default parameters. Furthermore, functional enrichment analysis was conducted to identify the significantly enriched Gene Ontology (GO) terms and metabolic pathways among the DEGs, with a significance threshold set at a *p*-value < 0.05 relative to the entire transcriptome background. GO functional enrichment analysis was performed using Goatools (0.6.5) [22], while KEGG pathway analysis was conducted using KOBAS (2.1.1) [23].

### 2.7. Gene Set Enrichment Analysis (GSEA)

To identify genes that may not show significant differences in overall expression level but have significant biological significance, we conducted Gene Set Enrichment Analysis (GSEA) [24] using the online tool provided by Majorbio Cloud Platform [25] (https://cloud.majorbio.com/page/tools/, accessed on 15 October 2022). Pathways with a *p*-value < 0.05, false discovery rate (FDR) < 0.25, and normalized enrichment score |NES| > 1 were selected for further analysis.

### 2.8. qRT-PCR

Random genes were selected to validate the RNA-seq data. Gene-specific primers were designed with NCBI Primer-BLAST and listed in Table S1. The qRT-PCR was performed by NovoStart^®^SYBR qPCR SuperMix plus regent (Novoprotein, Shanghai, China) and ABI 7500Fast Real-Time PCR System (ThermoFisher, Waltham, MA, USA) following the manufacturer’s protocol.

### 2.9. Economic Features Comparison

An additional set of 60 individuals from two experimental groups were reared on fresh mulberry leaves until the spinning stage. After five days of spinning, various economic features were measured, including the weight of the cocoon shell and the weight of the pupa. Subsequently, the cocoon shell ratio was calculated using the following formula: cocoon shell ratio = cocoon shell weight/(cocoon shell weight + pupa weight).

## 3. Results

### 3.1. Overview of the RNA-Seq Data

**Figure 1:**
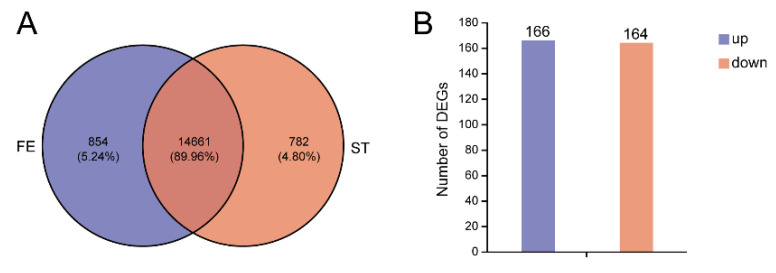
The number of identified genes (**A**) and differential expressed genes (**B**) in RNA-seq data. FE: feeding group; ST: starvation group.

A total of 40.7 Gb of clean data was obtained from the 6 samples. The mapping ratios of the clean reads are presented in Table S2. After aligning the reads to the silkworm genome, a total of 26,847 genes were identified, including 4368 novel genes (Table S3). All transcripts were integrated into a fa file named Data S1. Using DESeq2, we filtered 330 DEGs, comprising 166 up-regulated genes and 164 down-regulated genes (Figure 1 and Figure S1, and Table S4). At the same time, we carried out correlation and principal component analysis of the RNA-seq data, as shown in Figure S2.

### 3.2. Functional Annotation of DEGs

**Figure 2:**
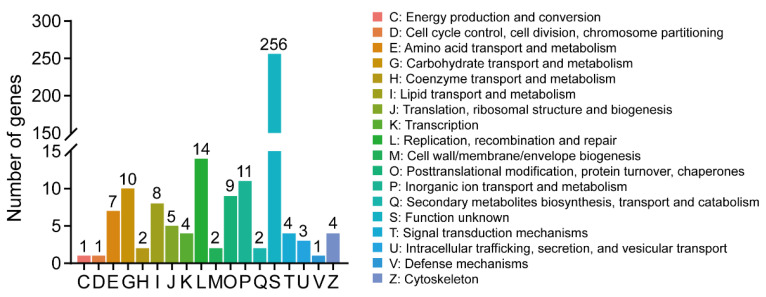
COG analysis of DEGs.

We utilized three different databases, namely COG, GO, and KEGG, to investigate the potential physiological functions of the differentially expressed genes (DEGs). Among the DEGs, eighteen COG classes were identified (Figure 2), with over 70% of the DEGs (256 genes) falling into the S category, indicating an unknown function. Additionally, several DEGs were found to be involved in other COG classes, such as L (replication, recombination, and repair) with 14 genes, P (inorganic ion transport and metabolism) with 11 genes, G (carbohydrate transport and metabolism) with 10 genes, O (posttranslational modification, protein turnover, chaperones) with 9 genes, I (lipid transport and metabolism) with 8 genes, and E (amino acid transport and metabolism) with 7 genes.

**Figure 3:**
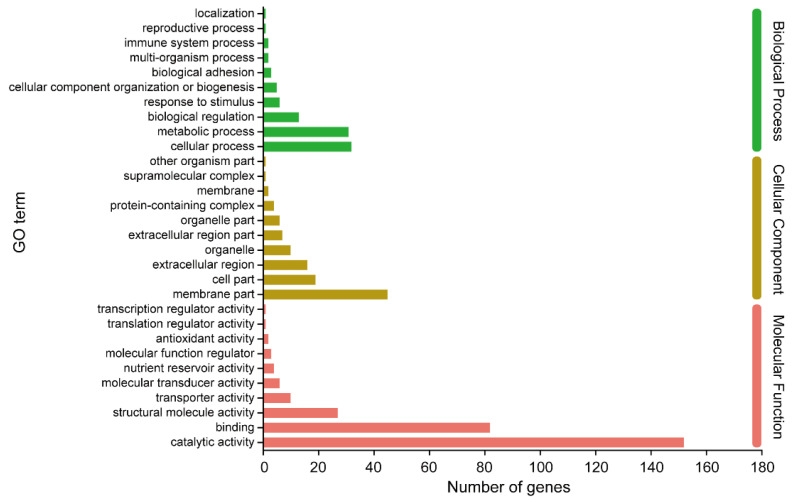
GO annotation of DEGs.

The GO annotation was categorized into three classes: Biological Process (BP), Cellular Component (CC), and Molecular Function (MF). Figure 3 illustrates the top 30 sub-classes of GO. In the Biological Process category, the most prevalent sub-classes were cellular process, metabolic process, and biological regulation. Furthermore, we observed that certain genes were involved in response to stimulus and immune system processes. Regarding the Cellular Component, the majority of DEGs were associated with the membrane part, cell part, and extracellular region. Within the Molecular Function class, which contained the largest number of DEGs, the top three sub-classes were catalytic activity, binding, and structural molecular activity, respectively. Additionally, the nutrient reservoir activity sub-class was also annotated, suggesting its potential involvement in response to starvation stimulus.

**Figure 4:**
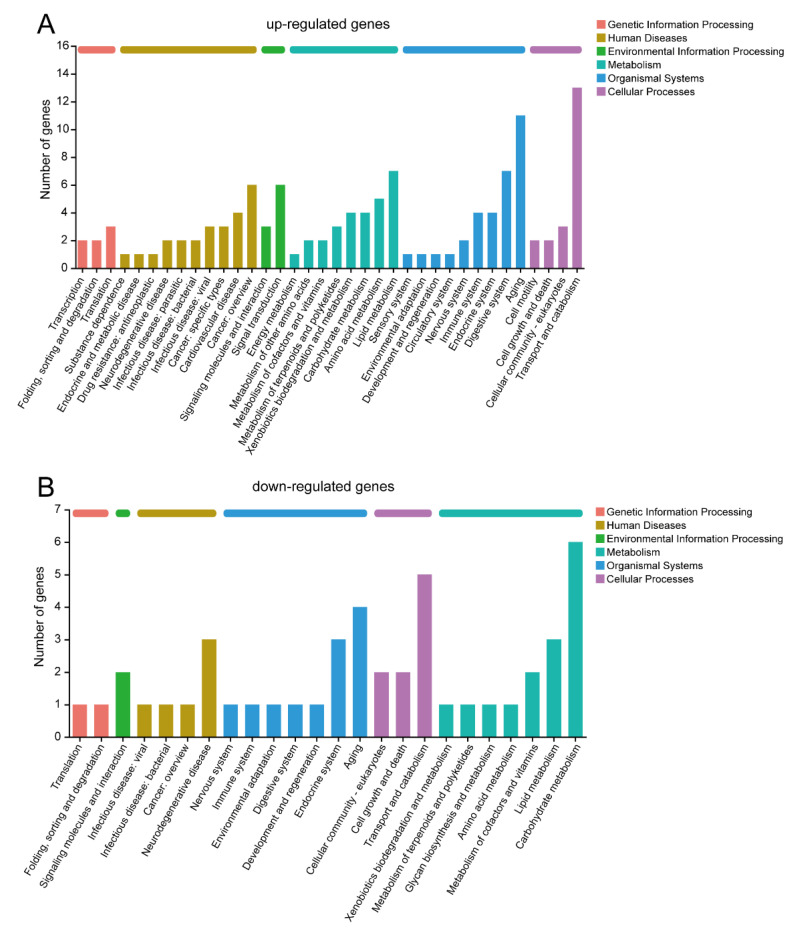
KEGG pathway annotation of DEGs. (**A**) up-regulated genes; (**B**) down-regulated genes.

In the KEGG pathway annotation, the up-regulated and down-regulated genes were analyzed separately (Figure 4). There are a total of six categories of KEGG pathways, and the majority of pathways were consistent between the up-regulated and down-regulated genes. In the Genetic Information Processing category, the DEGs were involved in translation and folding, sorting, and degradation. Within the Environmental Information Processing category, the DEGs participated in signaling molecules and interactions. The Cellular Processes category showed that cellular community-eukaryotes, cell growth and death, and transport and catabolism were common pathways shared by both up-regulated and down-regulated genes. The up-regulated genes exhibited a greater variety of Human Diseases pathways compared to the down-regulated genes, including involvement in cancer, neurodegenerative diseases, and infectious diseases. Notably, only the up-regulated genes were associated with endocrine and metabolic diseases, as well as drug-related pathways. The Metabolism category revealed the identification of nutrient metabolism pathways such as lipid, amino acid, and carbohydrate metabolism. Additionally, silkworm brain after starvation treatment displayed active pathways related to xenobiotics, terpenoids, and polyketides metabolism. The Aging pathway exhibited the highest number of DEGs within the Organism Systems category. Moreover, numerous DEGs were associated with the endocrine, digestive, immune, and nervous systems. Interestingly, only the up-regulated genes were involved in the sensory and circulatory systems.

### 3.3. GO and KEGG Enrichment Analysis

**Figure 5:**
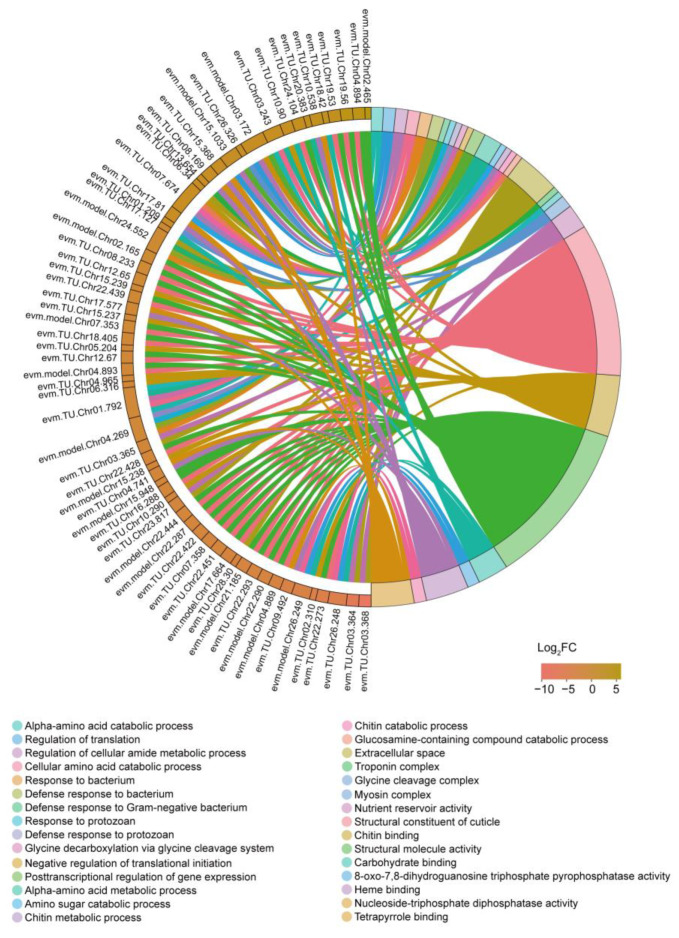
GO enrichment analysis of DEGs.

To further investigate the functions of DEGs, we performed GO and KEGG enrichment analyses. We identified a total of 47 significantly enriched GO terms with a *p*-value < 0.05 (Table S5), and a chord diagram was created to visualize the top 30 terms (Figure 5). Among the enriched terms, there were numerous associations with nutrient metabolism, including processes such as alpha-amino acid catabolic process, cellular amino acid catabolic process, alpha-amino acid metabolic process, nutrient reservoir activity, and carbohydrate binding. Notably, chitin metabolism terms were also prominently enriched, encompassing amino sugar catabolic process, chitin metabolic process, chitin catabolic process, glucosamine-containing compound catabolic process, structural constituent of cuticle, and chitin binding. Furthermore, immune-related terms showed enrichment, including response to bacterium, defense response to bacterium, defense response to Gram-negative bacterium, response to protozoan, and defense response to protozoan. Additionally, gene transcription and translation processes exhibited enrichment, with terms such as regulation of translation, negative regulation of translational initiation, posttranscriptional regulation of gene expression, 8-oxo-7,8-dihydroguanosine triphosphate pyrophosphatase activity, and nucleoside-triphosphate diphosphatase activity being enriched.

**Figure 6:**
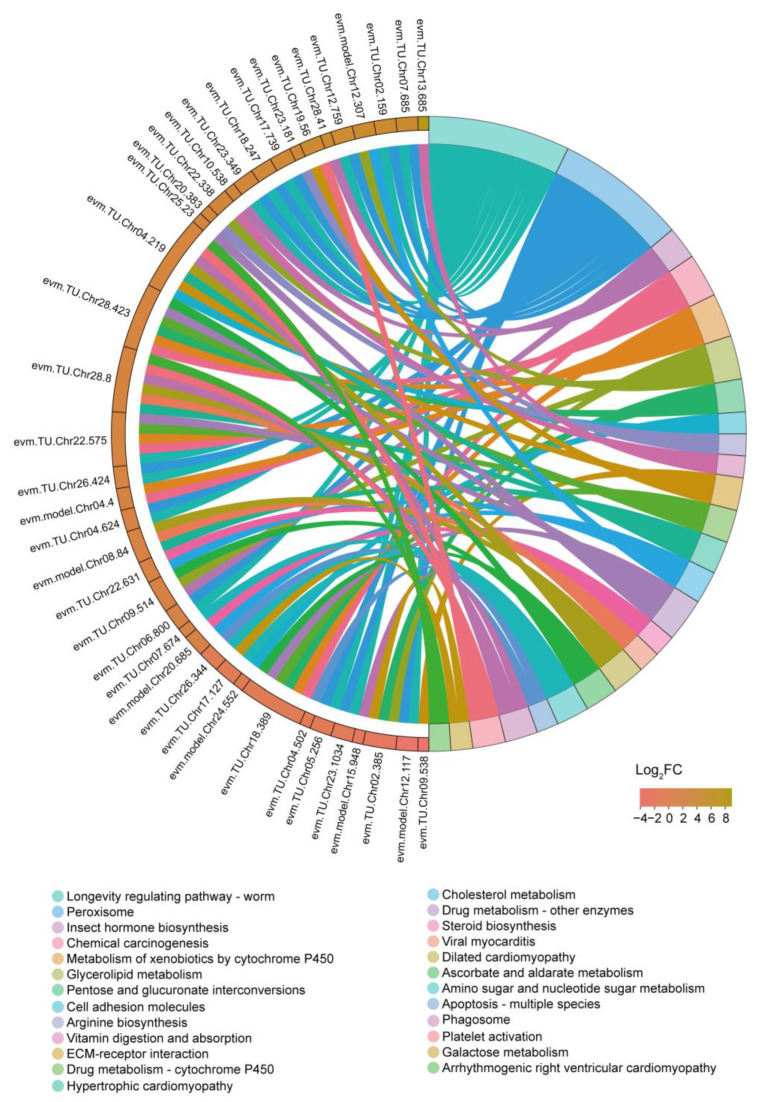
KEGG pathway enrichment analysis of DEGs.

We performed KEGG pathway enrichment analysis and identified 25 pathways that showed enrichment with a *p*-value < 0.05 (Figure 6 and Table S6). Consistent with the enrichment of GO terms, we observed the enrichment of numerous KEGG pathways related to nutrient metabolism. These included pathways such as glycerolipid metabolism, pentose and glucuronate interconversions, arginine biosynthesis, vitamin digestion and absorption, ascorbate and aldarate metabolism, and galactose metabolism. Hormones play a crucial role in regulating insect physiological activities, and we found three enriched pathways related to hormone biosynthesis, cholesterol metabolism, and steroid biosynthesis. Interestingly, the longevity regulating pathway-worm was also enriched following the starvation treatment. Furthermore, we observed enrichment of several disease-associated pathways in our data.

### 3.4. GSEA

GSEA (Gene Set Enrichment Analysis) is an algorithm that performs differential expression analysis at the level of gene sets [24]. GO provides functional annotations for genes, KEGG offers information about biological pathways and networks, and GSEA allows for the analysis of gene expression patterns within predefined gene sets to uncover relevant biological pathways or functions associated with specific experimental conditions. Since the MSigDB database does not cover silkworm data, we used the annotation result of our RNA-seq data as the prior gene set. In total, 90 gene sets were enriched, including 40 GO terms and 50 KEGG pathways (Table 1). The gene sets marked in red represent the terms or pathways that we have already analyzed through GO and KEGG enrichment. We observed that only 12 gene sets were duplicated, indicating that the results of GSEA can complement the enrichment analysis of DEGs. Upon examining the descriptions of the gene sets, we found that many of them were involved in nucleic acid metabolism, immune response and diseases, sugar and energy metabolism, amino acid and protein metabolism, as well as cellular communication.

### 3.5. Results of qRT-PCR

A total of 10 genes were selected from the RNA-seq data for qRT-PCR analysis, and the corresponding results are presented in Table 2. The majority of the results exhibited consistent trends, and the correlation coefficient between the fold change (FC) values obtained from qPCR and RNA-seq was determined to be 0.9786. This high correlation coefficient suggests that our results are reliable and trustworthy.

### 3.6. The Effect of Starvation on Silkworm Economic Features

**Figure 7:**
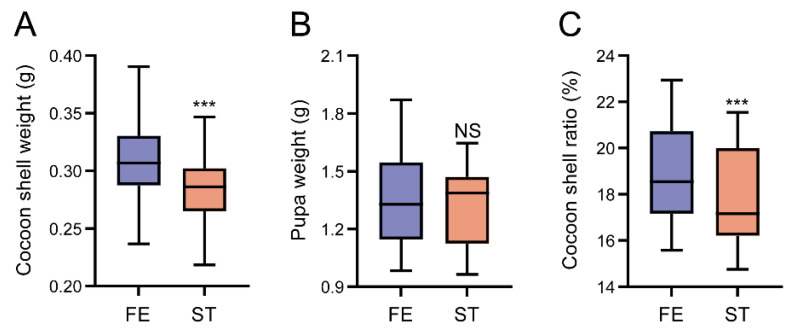
Economic features of silkworm. (**A**) Cocoon shell weight, (**B**) pupa weight, (**C**) cocoon shell ratio. FE: feeding group; ST: starvation group. ***, Student’s *t*-test *p*-value < 0.001; NS, no significance.

Starvation has severe influence on organism development. The economic features of starvation silkworm were observed and shown in Figure 7. After starvation treatment, the cocoon shell weight (Figure 7A) and cocoon shell ratio (Figure 7C) decreased significantly, while the pupa weight (Figure 7B) decreased with no significance.

## 4. Discussion

Starvation is a state characterized by a significant deficiency in energy intake, which falls below the level necessary to sustain an organism’s life. In humans, prolonged starvation can lead to permanent damage to essential organs [26] and, ultimately, result in death. The brain, serving as the central hub of the nervous and endocrine systems, holds utmost significance in animals. In order to shed light on the impact of starvation on the brain of animals, we conducted a comprehensive transcriptome analysis utilizing the silkworm as a model organism.

Following a period of starvation, notable changes were observed in genes associated with nutrient metabolism, particularly in energy metabolism. The brain, being a highly energy-demanding organ responsible for processing and managing information flow [27], is particularly vulnerable to the effects of energy deficiency. Consequently, an inadequate energy supply may have detrimental effects on the brain’s normal physiological functioning. Concurrently, alterations were detected in cellular pathways, including the extracellular space and ECM-receptor interaction. The stability of cellular structures plays a crucial role in organ functionality, indicating that starvation may potentially induce cellular damage in the brain of silkworms. Moreover, an enrichment of pathways related to chitin and cuticle functions was observed post-starvation. In insects, the collaboration between chitin and cuticle proteins is essential for maintaining organ stability, such as in the epidermis [28], peritrophic membrane [29], and spinning duct [30]. These findings further substantiate the notion that starvation can significantly impact the stability of the silkworm brain.

The silkworm brain serves as the central hub of the endocrine system, playing a pivotal role in synthesizing and secreting numerous hormones that regulate the growth and development of silkworms [31]. For instance, the brain secretes the prothoracicotropic hormone (PTTH), which then travels to the prothoracic glands to initiate the biosynthesis of ecdysone, a crucial hormone involved in the molting process [32]. Previous studies have demonstrated that prolonged periods of starvation in silkworm larvae can elevate the production rate of ecdysteroids, thereby enhancing their survival rate [33]. In our study, we also observed changes in hormone-related pathways following starvation, suggesting that food deprivation can potentially lead to developmental abnormalities in silkworms. It is widely recognized that development and longevity are closely intertwined across different species [34]. Notably, we observed an enrichment of the longevity-regulating pathway in our data (Figure 6). Research conducted on various model organisms has shown that starvation, in the absence of malnutrition, can extend lifespan and mitigate age-related diseases [35], which aligns with our findings.

Nucleic acids, encompassing DNA and RNA, are fundamental molecules for life. DNA serves as the primary repository, replicator, and transmitter of genetic information, while RNA plays a crucial role in protein synthesis [36]. Following exposure to starvation, numerous pathways involved in nucleic acid metabolism underwent changes in the silkworm brain (Figure 5 and Figure 6, Table 1). In a study on blue crabs, researchers observed a decrease in nucleic acid concentration after starvation and proposed that it could serve as a reliable and sensitive indicator of nutritional stress [37]. Based on this, we hypothesized that the nucleic acid concentration is altered following starvation treatment. Additionally, disruptions in nucleic acid metabolism may lead to modifications in other pathways, including those associated with immune response and diseases, as evidenced by our results (Figure 5 and Figure 6, Table 1). Recent studies have revealed the existence of a diverse population of immune cells residing in the dura and meninges surrounding the brain [38,39], indicating that the brain possesses its own reservoir of immune cells. Starvation has been demonstrated to impact organismal immune responses in various species [40,41,42,43]. Consistent with our findings, a plethora of immune-related pathways were enriched, suggesting that starvation can influence the immune system in the silkworm brain.

We have observed significant changes in the transcriptome data of several well-known genes associated with appetite regulation. Dopamine, a versatile neurotransmitter crucial for animal behavior [44,45], is synthesized through the activity of tyrosine hydroxylase, an enzyme that acts as a pivotal step in dopamine biosynthesis [46]. A recent study on honeybees has demonstrated that the seeking of food is modulated by transient activation of dopaminergic signaling in the honey bee brain [47]. Our previous research has also shown that the overexpression of tyrosine hydroxylase in the silkworm brain enhances foraging behavior in silkworm larvae [48]. In this current study, we observed a decrease in the expression of the tyrosine hydroxylase gene in the starvation group, providing further evidence for the involvement of tyrosine hydroxylase in the regulation of feeding behavior in silkworms.

In summary, our study involved a comprehensive transcriptome analysis of the silkworm brain in response to starvation treatment. The results shed light on substantial modifications observed in various pathways associated with crucial biological processes. Firstly, pathways related to tissue structure underwent notable changes, suggesting potential alterations in the organization and integrity of brain tissues after starvation. Secondly, hormone metabolism pathways exhibited significant modifications, indicating disruptions in the synthesis, secretion, and regulation of hormones crucial for various physiological processes. Additionally, pathways associated with longevity, a critical aspect of an organism’s lifespan, were also affected, suggesting that starvation may have implications for the aging process in silkworms. Moreover, significant alterations were observed in pathways related to nucleic acid metabolism, suggesting potential dysregulation in DNA and RNA processes, which are fundamental to genetic information storage and protein synthesis. Furthermore, pathways associated with immune response and diseases were also significantly impacted, indicating potential consequences for the silkworm’s immune system and overall health status. Taken together, our findings provide valuable insights into the wide-ranging effects of starvation on the transcriptomic profile of the silkworm brain, highlighting the intricate interplay between nutrient availability and various biological processes.

## Figures and Tables

**Table 1:** Gene set enrichment analysis results in RNA-seq data. ES, enrichment score; NES, normalized enrichment score.

Gene Set	Description	Leading Edge/Size	ES	NES	*p*-Value	FDR
GO:0009166	nucleotide catabolic process	12/19	0.75	1.99	0	0.014
GO:1901292	nucleoside phosphate catabolic process	12/19	0.75	1.96	0	0.014
GO:0042742	defense response to bacterium	10/18	0.70	1.83	0	0.020
GO:0006955	immune response	15/34	0.61	1.81	0	0.020
GO:0009150	purine ribonucleotide metabolic process	28/50	0.57	1.83	0	0.021
GO:0098542	defense response to other organism	10/19	0.68	1.81	0.0016	0.022
GO:0009607	response to biotic stimulus	10/19	0.68	1.81	0.0032	0.022
GO:0006753	nucleoside phosphate metabolic process	50/101	0.49	1.79	0	0.022
GO:0002376	immune system process	16/36	0.61	1.86	0	0.023
GO:0009117	nucleotide metabolic process	50/101	0.49	1.79	0	0.023
GO:1901135	carbohydrate derivative metabolic process	75/149	0.49	1.83	0	0.023
GO:0045087	innate immune response	15/30	0.62	1.84	0.0015	0.024
GO:0043207	response to external biotic stimulus	10/19	0.68	1.78	0.0016	0.025
GO:0006091	generation of precursor metabolites and energy	21/33	0.63	1.87	0	0.026
GO:0046434	organophosphate catabolic process	13/27	0.66	1.88	0	0.027
GO:0009617	response to bacterium	10/18	0.70	1.84	0	0.027
GO:0006720	isoprenoid metabolic process	11/16	0.71	1.76	0.0016	0.028
GO:0019693	ribose phosphate metabolic process	35/60	0.57	1.89	0.0014	0.030
GO:0051707	response to other organism	10/19	0.68	1.76	0.0032	0.030
GO:0009152	purine ribonucleotide biosynthetic process	24/45	0.55	1.74	0.0044	0.035
GO:0044455	mitochondrial membrane part	27/42	0.60	1.88	0	0.011
GO:0098800	inner mitochondrial membrane protein complex	19/30	0.64	1.90	0	0.014
GO:0098798	mitochondrial protein complex	30/50	0.56	1.79	0.0015	0.026
GO:0044429	mitochondrial part	70/135	0.45	1.71	0	0.069
GO:1990204	oxidoreductase complex	11/16	0.66	1.64	0.0047	0.12
GO:0044815	DNA packaging complex	6/24	0.57	1.59	0.024	0.17
GO:0005615	extracellular space	21/96	0.42	1.52	0.0094	0.17
GO:0098796	membrane protein complex	86/163	0.39	1.51	0.0053	0.18
GO:0031090	organelle membrane	63/126	0.41	1.52	0.0053	0.19
GO:0000786	nucleosome	6/22	0.58	1.56	0.025	0.19
GO:0044421	extracellular region part	22/103	0.42	1.53	0.0080	0.20
GO:0031966	mitochondrial membrane	28/56	0.47	1.54	0.0030	0.20
GO:0019866	organelle inner membrane	15/38	0.48	1.47	0.036	0.21
GO:0005743	mitochondrial inner membrane	15/38	0.48	1.46	0.042	0.21
GO:0005623	cell	27/50	0.45	1.47	0.032	0.22
GO:0032993	protein-DNA complex	6/23	0.53	1.44	0.043	0.23
GO:0042302	structural constituent of cuticle	35/139	0.64	2.42	0	0
GO:0005198	structural molecule activity	64/414	0.44	1.82	0	0.06
GO:0005179	hormone activity	27/49	−0.51	−1.81	0	0.13
GO:0019200	carbohydrate kinase activity	12/32	0.57	1.68	0.0046	0.21
MAP04540	Gap junction	27/57	−0.52	−1.66	0.0015	0.042
MAP04215	Apoptosis—multiple species	7/19	−0.60	−1.57	0.024	0.067
MAP04144	Endocytosis	70/149	−0.38	−1.44	0.019	0.14
MAP04310	Wnt signaling pathway	49/87	−0.42	−1.48	0.0069	0.10
MAP04012	ErbB signaling pathway	24/58	−0.45	−1.50	0.018	0.11
MAP04024	cAMP signaling pathway	50/149	−0.36	−1.38	0.013	0.16
MAP04013	MAPK signaling pathway—fly	47/129	−0.37	−1.40	0.022	0.17
MAP04071	Sphingolipid signaling pathway	37/77	−0.44	−1.50	0.014	0.17
MAP03050	Proteasome	28/38	−0.64	−1.98	0	0.0011
MAP04141	Protein processing in endoplasmic reticulum	72/131	−0.50	−1.87	0	0.0015
MAP03060	Protein export	13/23	−0.65	−1.76	0.0078	0.0095
MAP03010	Ribosome	85/121	0.32	1.40	0.016	0.12
MAP03020	RNA polymerase	16/76	0.37	1.43	0.022	0.15
MAP03410	Base excision repair	5/22	0.49	1.48	0.044	0.21
MAP05012	Parkinson disease	67/128	−0.53	−1.96	0	0.00078
MAP05322	Systemic lupus erythematosus	14/22	−0.69	−1.84	0.0016	0.0046
MAP05034	Alcoholism	35/74	−0.50	−1.72	0	0.015
MAP05410	Hypertrophic cardiomyopathy (HCM)	22/62	0.48	1.77	0	0.046
MAP04934	Cushing syndrome	36/84	−0.44	−1.58	0.0044	0.054
MAP05414	Dilated cardiomyopathy (DCM)	14/60	0.44	1.65	0.0031	0.066
MAP04932	Non-alcoholic fatty liver disease (NAFLD)	65/155	−0.41	−1.54	0.0038	0.069
MAP05203	Viral carcinogenesis	65/123	−0.40	−1.49	0.010	0.081
MAP05030	Cocaine addiction	16/29	−0.53	−1.50	0.034	0.082
MAP05226	Gastric cancer	29/89	−0.39	−1.36	0.049	0.17
MAP05016	Huntington disease	98/291	−0.32	−1.29	0.030	0.19
MAP00900	Terpenoid backbone biosynthesis	14/28	0.67	1.92	0	0.0071
MAP00220	Arginine biosynthesis	8/25	−0.62	−1.88	0	0.0073
MAP00030	Pentose phosphate pathway	14/26	0.67	1.84	0	0.012
MAP00190	Oxidative phosphorylation	57/126	0.47	1.76	0	0.022
MAP00640	Propanoate metabolism	18/28	0.59	1.71	0.0046	0.037
MAP00981	Insect hormone biosynthesis	5/36	−0.49	−1.66	0.0027	0.039
MAP00051	Fructose and mannose metabolism	16/31	0.56	1.61	0.011	0.092
MAP00052	Galactose metabolism	12/38	0.51	1.55	0.0094	0.12
MAP00790	Folate biosynthesis	23/44	0.49	1.56	0.016	0.13
MAP00380	Tryptophan metabolism	12/32	0.51	1.49	0.030	0.14
MAP00010	Glycolysis/Gluconeogenesis	24/50	0.46	1.49	0.015	0.15
MAP00040	Pentose and glucuronate interconversions	22/68	0.44	1.50	0.022	0.16
MAP00280	Valine, leucine and isoleucine degradation	12/39	0.47	1.44	0.048	0.17
MAP00062	Fatty acid elongation	9/28	0.51	1.45	0.037	0.17
MAP04723	Retrograde endocannabinoid signaling	43/85	−0.52	−1.83	0	0.022
MAP04916	Melanogenesis	35/50	−0.52	−1.66	0.0059	0.057
MAP04714	Thermogenesis	86/198	−0.43	−1.69	0	0.067
MAP04912	GnRH signaling pathway	28/54	−0.46	−1.51	0.028	0.13
MAP04728	Dopaminergic synapse	33/66	−0.46	−1.53	0.0059	0.13
MAP04927	Cortisol synthesis and secretion	22/38	−0.48	−1.48	0.028	0.14
MAP04928	Parathyroid hormone synthesis, secretion and action	25/47	−0.47	−1.49	0.026	0.14
MAP04721	Synaptic vesicle cycle	39/72	−0.42	−1.44	0.018	0.14
MAP04970	Salivary secretion	25/55	−0.43	−1.43	0.038	0.15
MAP04740	Olfactory transduction	18/28	−0.51	−1.46	0.044	0.15
MAP04926	Relaxin signaling pathway	34/77	−0.44	−1.54	0.016	0.16

**Table 2:** qRT-PCR quantification of expression levels of selected genes and comparison with their RNA-seq fold change.

Gene ID	qPCR FC	RNA-Seq FC	Gene ID	qPCR FC	RNA-Seq FC
evm.TU.Chr26.279	0.91	1.36	evm.TU.Chr01.298	0.38	0.52
evm.TU.Chr24.417	0.42	0.93	evm.TU.Chr04.602	0.58	0.65
evm.TU.Chr06.225	0.74	0.66	evm.model.Chr10.490	0.68	0.95
evm.TU.Chr11.555	2.35	1.16	evm.TU.Chr02.21	0.67	0.92
evm.TU.Chr22.484	0.99	1.13	evm.TU.Chr12.759	8.13	11.07

## Data Availability

The data presented in this study are available on request from the first author.

## Supplementary Materials

The following supporting information can be downloaded at: https://www.mdpi.com/article/10.3390/insects14070658/s1. Figure S1: The volcano plot (A) and scatter plot (B) of DEGs; Figure S2: Correlation and principal components analysis of RNA-seq data. Table S1: Primers used in this study; Table S2: Mapping ratio of reads data. Table S3: All identified genes in RNA-seq. Table S4: DEGs identified in RNA-seq. Table S5: GO enrichment analysis of DEGs. Table S6: KEGG enrichment analysis of DEGs. Data S1: All transcripts identified in this data.
