# Supplementary figures and images for: Transcriptomic Analysis of Starvation on the Silkworm Brain
Source: Insects. 2023 Jul 24;14(7):658. doi: 10.3390/insects14070658 (PMC10380768; doi:10.3390/insects14070658)

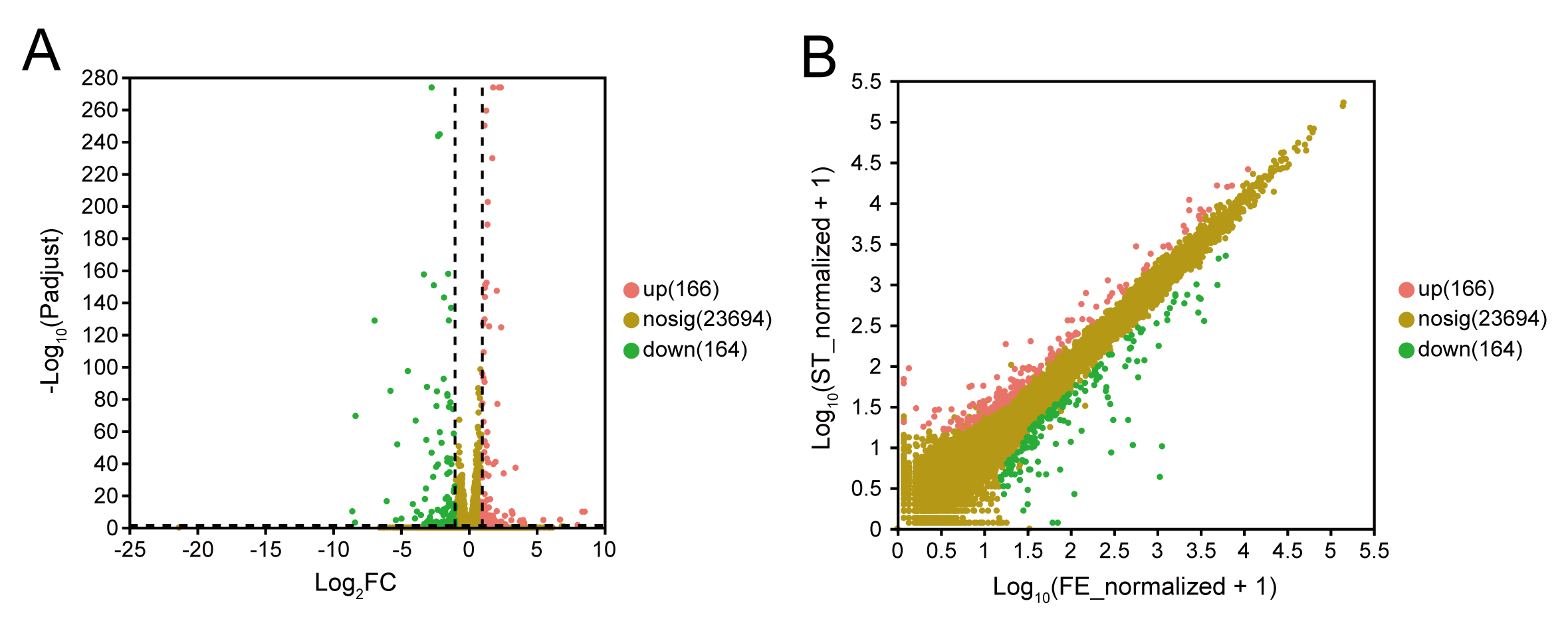

Supplement: Supplementary file 1 [file insects-14-00658-s001.zip › Figure S1.tif]

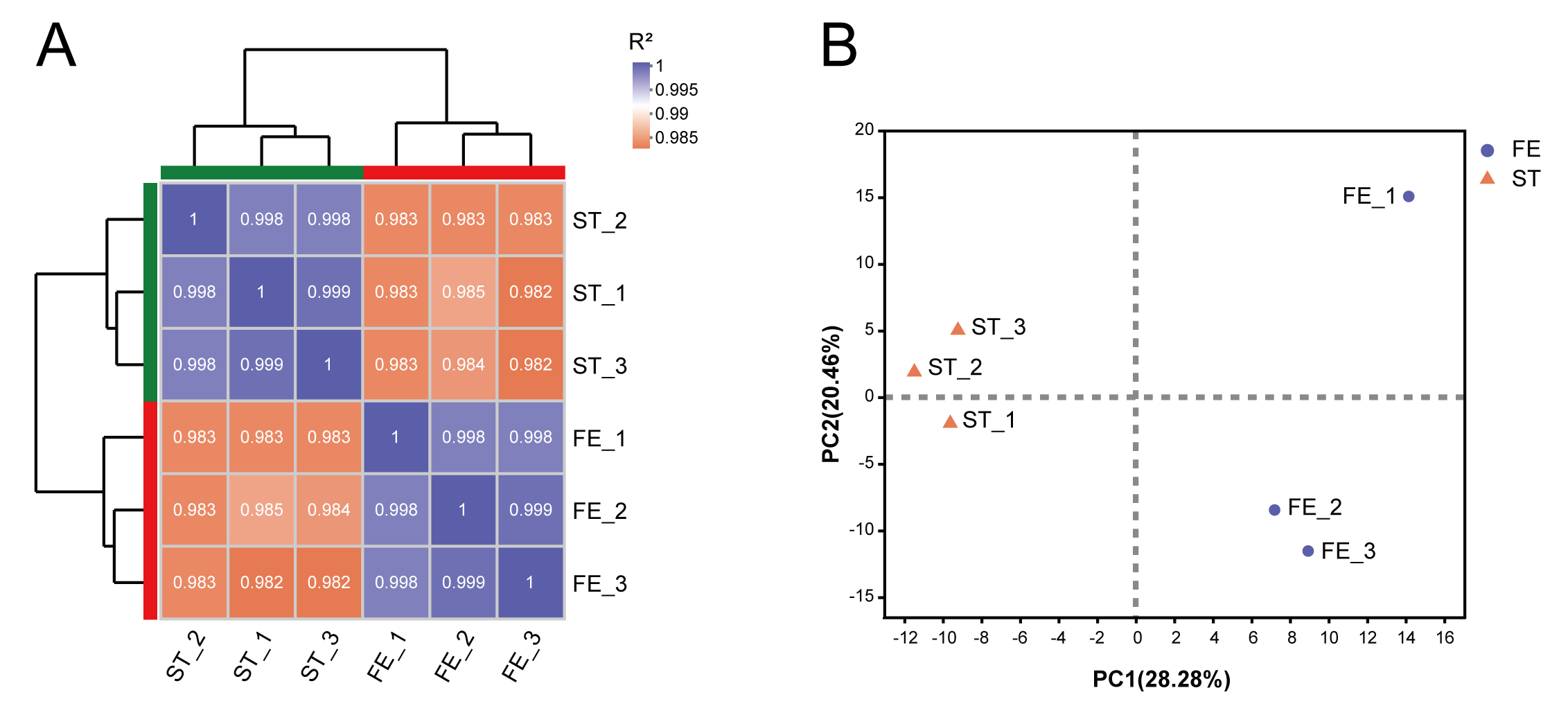

Supplement: Supplementary file 1 [file insects-14-00658-s001.zip › Figure S2.tif]
